# Structure-function coupling in white matter uncovers the abnormal brain connectivity in Schizophrenia

**DOI:** 10.1038/s41398-023-02520-4

**Published:** 2023-06-21

**Authors:** Jiajia Zhao, Chu-Chung Huang, Yajuan Zhang, Yuchen Liu, Shih-Jen Tsai, Ching-Po Lin, Chun-Yi Zac Lo

**Affiliations:** 1 Institute of Science and Technology for Brain-Inspired Intelligence, Fudan University, Shanghai, China; 2 Shanghai Key Laboratory of Brain Functional Genomics (Ministry of Education), Affiliated Mental Health Center (ECNU), Institute of Cognitive Neuroscience, School of Psychology and Cognitive Science, East China Normal University, Shanghai, China; 3 Shanghai Changning Mental Health Center, Shanghai, China; 4 School of Biomedical Engineering, ShanghaiTech University, Shanghai, China; 5 Department of Psychiatry, Taipei Veterans General Hospital, Taipei, Taiwan; 6 Division of Psychiatry, Faculty of Medicine, National Yang Ming Chiao Tung University, Taipei, Taiwan; 7 Institute of Brain Science, National Yang Ming Chiao Tung University, Taipei, Taiwan; 8 Institute of Neuroscience, National Yang Ming Chiao Tung University, Taipei, Taiwan; 9 Department of Education and Research, Taipei City Hospital, Taipei, Taiwan; 10 Department of Biomedical Engineering, Chung Yuan Christian University, Taoyuan, Taiwan

**Keywords:** Neuroscience, Physiology

## Abstract

Schizophrenia is characterized by dysconnectivity syndrome. Evidence of widespread impairment of structural and functional integration has been demonstrated in schizophrenia. Although white matter (WM) microstructural abnormalities have been commonly reported in schizophrenia, the dysfunction of WM as well as the relationship between structure and function in WM remains uncertain. In this study, we proposed a novel structure-function coupling measurement to reflect neuronal information transfer, which combined spatial-temporal correlations of functional signals with diffusion tensor orientations in the WM circuit from functional and diffusion magnetic resonance images (MRI). By analyzing MRI data from 75 individuals with schizophrenia (SZ) and 89 healthy volunteers (HV), the associations between structure and function in WM regions in schizophrenia were examined. Randomized validation of the measurement was performed in the HV group to confirm the capacity of the neural signal transferring along the WM tracts, referring to quantifying the association between structure and function. Compared to HV, SZ showed a widespread decrease in the structure-function coupling within WM regions, involving the corticospinal tract and the superior longitudinal fasciculus. Additionally, the structure-function coupling in the WM tracts was found to be significantly correlated with psychotic symptoms and illness duration in schizophrenia, suggesting that abnormal signal transfer of neuronal fiber pathways could be a potential mechanism of the neuropathology of schizophrenia. This work supports the dysconnectivity hypothesis of schizophrenia from the aspect of circuit function, and highlights the critical role of WM networks in the pathophysiology of schizophrenia.

## Introduction

Schizophrenia is a severe psychiatric disorder associated with dysconnectivity in the brain [1], showing abnormal communication in the brain networks via diffusion and functional magnetic resonance imaging (fMRI) studies [2–5]. Widespread WM microstructural abnormalities have been revealed using diffusion tensor imaging (DTI), indicating that WM alterations may play a critical role in the etiology of schizophrenia [6–8]. On the other hand, recent evidence has indicated that functional activity in WM has physiological significance, and the resting-state fMRI in WM manifests an intrinsic functional organization [9–11]. Previous studies have observed that fMRI activation in specific WM bundles can be modulated by different tasks [12–15]. These evidences provide a new perspective to investigate the WM dysfunction in schizophrenia [11, 16–18].

To probe the functional activity of the brain WM structure, researchers proposed the functional correlation tensor (FCT) approach, which computes the magnitude and orientation of functional activation in WM based on fMRI signals [19, 20]. FCT in WM was shown to be affected by the level of anesthesia, reflecting the underlying neural activity [21]. The directional characteristics of FCT have shown the ability to depict fiber bundles such as corpus callosum and optical radiation using task or resting-state fMRI, which is similar to those reconstructed by DTI techniques [19, 20]. Moreover, FCT has been used to predict obesity [22], survival time for patients with brain tumors [23], and classification and diagnosis of diseases [24, 25]. These studies demonstrated that FCT can be used to reveal how information is transmitted along WM tracts based on resting-state fMRI, thereby providing a perspective beyond empirical functional connectivity.

Considering the dysconnectivity hypothesis and widely reported abnormal WM connectivity issues in schizophrenia, previous studies have explored abnormal structure-function coupling with connectivity correlations [26, 27]. However, the alteration of the association between structural and functional connectivity was not consistent between different functional networks. These changes have also been found to be related to clinical symptoms and illness duration in schizophrenia [26, 28–31]. In most studies, structural characteristics were measured using diffusion tractography based on the diffusion tensor (DT) reconstructed in WM and functional connectivities were estimated separately using fMRI in gray matter (GM). Because the nature of diffusion tractography-based structural connections and statistic-based functional connectivity linking distinct GM regions are quite different, direct integration of these traits provides limited insights regarding the structural connections underlying functional interactions [32]. Taking advantage of the fact that DT can provide anatomical information of WM with respect to fiber orientation, while FCT can depict the local functional anisotropy of WM, the combination of these two techniques may allow us to explore the function of WM connections in schizophrenia.

In this study, we combined the FCT and DT by accounting the local functional anisotropy and microstructural orientations to characterize the structure-function relationship in the WM tracts. Given the abnormality of functional networks as well as structure-function integration in schizophrenia, we hypothesized that neural signals along the neural tracts would be altered in individuals with schizophrenia. We therefore analyzed resting-state fMRI and diffusion weighted imaging data from 75 individuals with schizophrenia (SZ) and 89 healthy volunteers (HV). The FCT-DT consistency in the WM tracts was first validated in HV group. Subsequently, the measurement was then used to directly explore the structure-function coupling in schizophrenia. We predicted that the FCT-DT consistency in the neural tracts would be altered in SZ, compared to HV. The associations between the FCT-DT consistency in the WM tracts and psychotic symptoms in SZ were also tested.

## Methods

### Participants

A total of 207 participants were recruited: individuals with schizophrenia, SZ (*n* = 103) and healthy volunteers, HV (*n* = 104). Participants were recruited from the Taipei Veteran General Hospital, Taiwan, which is a part of Taiwan Aging and Mental Illness cohort. SZ was diagnosed according to the Diagnostic and Statistical Manual of Mental Disorders-IV, DSM-IV criteria for schizophrenia. The severity of symptoms was evaluated using the Positive and Negative Syndrome Scale (PANSS), including the positive scale (7 items), the negative scale (7 items), and the general psychopathology scale (16 items) [33]. All SZ were medicated and the average antipsychotic dose was 539 mg/d in chlorpromazine equivalents (The detailed information for each subject were provided in Supplementary Table 1). HV group had no history of neurological or psychiatric disorders confirmed by the Mini-International Neuropsychiatric Interview, MINI [34]. This study was approved by the Institutional Review Board of Taipei Veterans General Hospital, Taipei, Taiwan, and written informed consent was obtained from all participants. All participants also underwent the Mini-Mental State Examination (MMSE) test to assess global cognitive performance [35]. Participants who had a history of substance abuse, mental retardation, systemic medical or neurological disorders, brain trauma, unstable psychotic symptoms, or adjustment to psychotropic medications within 3 months before the study were excluded. Following the imaging quality control procedures of diffusion/functional image data [36], 43 subjects were excluded due to incompleteness of the imaging data, motion artefacts, poor signal-to-noise ratio or poor gray/white contrast (more details on participant exclusion criteria were described in Supplementary method). Finally, the final sample included 75 SZ and 89 HV in this study (Table 1).

**Table 1:** Demographic and clinical characteristics of subjects.

Characteristic	SZ Mean ± S.D.	HV Mean ± S.D.	*P* value
Number	75	89	
Sex (F/M)	38/37	50/39	0.38^a^
Age	42.44 ± 11.04	39.61 ± 12.62	0.10^b^
Education	13.98 ± 2.59	14.69 ± 2.68	0.13^b^
MMSE	27.77 ± 1.97	28.58 ± 1.28	0.002^b^
Illness Duration	16.39 ± 9.81		
Dose (CPZ)^c^	539 ± 405	–	–
PANSS Score			
Positive	9.45 ± 2.88	–	–
Negative	9.24 ± 2.52	–	–
General	20.47 ± 5.18	–	–
Total	39.16 ± 9.10	–	–

^a^Chi-squared test.

^b^Two-sample *t* test.

^c^Chlorpromazine equivalent doses.

### Image acquisition

MRI data was acquired using a 3 T MR system (Siemens Magnetom Tim Trio, Erlangen, Germany) at National Yang Ming Chiao Tung University. An anatomical T1-weighted image was acquired with a sagittal 3D magnetization-prepared rapid gradient echo (MPRAGE) sequence: repetition time (TR) = 2530 ms, echo time (TE) = 3.5 ms, flip angle = 7°, inversion time = 1100 ms, FOV = 256 × 256 mm and voxel size = 1 × 1 × 1 mm^3^. Rs-fMRI data were acquired while subjects were lying quietly with their eyes closed in the scanner, using a gradient echo-planar imaging sequence with the following parameters: TR = 2500 ms, TE = 27 ms, flip angle = 77°, FOV = 220 × 220 mm^2^, matrix size = 64 × 64, voxel size = 3.44 × 3.44 × 3.4 mm^3^. A total of 200 EPI images were acquired along the AC–PC plane. Diffusion-weighted MRI data were acquired with single-shot spin-echo echo-planar imaging (SE-EPI) sequence: TR = 11000 ms, TE = 104 ms, FOV = 128×128 mm^2^, voxel size = 2 × 2 × 2 mm^3^, 70 contiguous axial slices, 30 non-collinear gradient directions with a b value of 1000 s/mm^2^ and three additional null images (b = 0 s/mm^2^) as reference images with NEX = 3.

### Image preprocessing

All MR data were processed identically. Functional images were preprocessed using AFNI (https://afni.nimh.nih.gov) and FSL (https://fsl.fmrib.ox.ac.uk/), including removal of the first ten time points; slice-timing correction; motion correction to the first volume with rigid-body alignment; obliquity transform to the structural MR image; wavelet despike [37]; multiple regression of six motion parameters and their first derivatives, cerebrospinal fluid (CSF), respiratory and cardinal signals from the fMRI time series data; temporally filtering with bandpass 0.01–0.1 Hz; intensity normalization to a whole brain median of 1000 [37, 38]; spatial smoothing within functional mask with a 6-mm at full-width at half-maximum Gaussian kernel; and co-registration to the diffusion MRI b0 image along with T1-weighted images. Subjects were excluded if any of the six head motion parameters estimated during movement correction were greater than 2 mm translation or 2 degrees rotation. Diffusion-weighted images were implemented by FSL and MRtrix3 [39] (https://www.mrtrix.org/). First, eddy current distortions and head movements were corrected using affine registration to the null image. The diffusion tensors were then fitted to the DWI using weighted least-squares estimation, and the fractional anisotropy (FA) of each voxel was also calculated.

### Construction of functional correlation tensor

For each voxel, the FCT was constructed to characterize the local profiles of temporal correlation between that voxel and its neighbors. According to Ding et al., The FCT represented as follows can be calculated from the spatial relationship of physical distances and temporal correlations of functional activity [20]:

$$FCT = \left[ {\begin{array}{*{20}{c}} {T_{xx}} & {T_{xy}} & {T_{xz}} \\ {T_{xy}} & {T_{yy}} & {T_{yz}} \\ {T_{xz}} & {T_{yz}} & {T_{zz}} \end{array}} \right]$$

Like the diffusion tensor, FCT contains functional correlation in each direction and measures local functional anisotropy in each voxel. The detailed mathematical solution for the FCT was shown in the Supplementary method. Here, we computed FCT of all voxels in WM using rs-fMRI for all subjects.

### WM tracts

First, the 48 tracts extracted from the JHU ICBM-81-DTI WM atlas were used as the region of interests [40]. For each individual, the null image in the DWI space was co-registered with the T1-weighted image; the T1-weighted image was normalized to the ICBM152 T1 template in the MNI space. To transfer the spaces between native DWI and MNI, we obtained the comprehensive transformation matrix and its inverse matrix. The inverse transformation matrix was used to apply the WM atlas from the MNI space to the native DWI space via nearest-neighbor interpolation [41]. In this study, we focused on cerebral regions, excluding the cerebellum and pons. As a result, 40 WM tracts were used to study the differences between SZ and HV groups in the structure-function coupling. The WM mask was generated with the FA value greater than 0.2. To avoid the potential influence of GM signals on WM signals, a WM mask with FA value > 0.2 was used for the WM atlas in the native DWI space.

### Evaluation of structure-function coupling

**Fig. 1:**
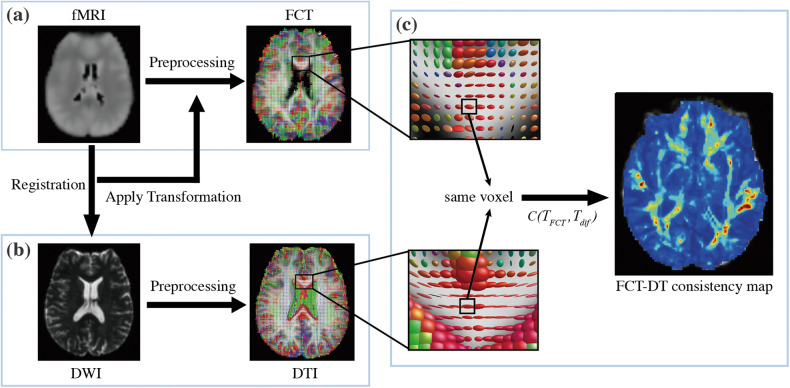
The flowchart of calculating FCT-DT consistency. **a** fMRI images were coregistered to DWI space using the transformation matrix acquired during the registration from fMRI images to DWI images, and then functional correlation tensor was computed. **b** The individual diffusion tensor was calculated in native DWI space derived from DWI images. **c** For each voxel, the FCT-DT consistency was estimated by measuring the Euclidean distance between the functional correlation tensor and diffusion tensor, which consequently generated the FCT-DT consistency map of the whole brain.

The structure-function coupling was evaluated by the consistency between FCT and DT (FCT-DT consistency) using the tensor difference equation modified from [42]. The measure of FCT-DT consistency, *C*(*T*_*FCT*_, *T*_*dif*_), for each voxel is defined as:

$$C\left( {T_{FCT},T_{dif}} \right) = \frac{1}{{\sqrt {trace\left[ {\left( {T_{FCT} - T_{dif}} \right)^2} \right]} }}FA_{FCT}\cdot FA_{dif}$$

where *T*_*FCT*_ and *T*_*dif*_ are functional correlation tensor and diffusion tensor in a specific voxel separately, normalized by the sum of all elements in the matrix to control the scales at the same level. Considering the anisotropy of tensor, the FA values were used as weighting factors. *FA*_*FCT*_ and *FA*_*dif*_ are FA values of functional correlation tensor and diffusion tensor separately. In general, the higher the value of *C*, the more consistent the match between FCT and DT. In this study, individual FCT-DT consistency maps of WM tracts were measured to evaluate structure-function coupling in WM (Fig. 1).

### Validation of FCT-DT consistency

To validate the nonrandom organization of FCT-DT consistency in the WM tracts, the mean FCT-DT consistency in voxels in a specific WM tract was compared to the mean FCT-DT consistency in voxels from GM (more details were described in the Supplementary method). Besides, in order to examine the internal consistency of FCT-DT consistency, the HV group was randomly divided into two halves and repeated 5000 times. The subgroup comparisons in FCT-DT consistency were made for all WM tracts.

### Statistical analysis

For demographic tests, a two-sample t-test was used to test group differences in age, education, and cognitive performance, while the chi-square test was used to test for sex differences. The significance level was established at *P* < 0.05 with FDR correction for randomized validation of the newly proposed measurement, FCT-DT consistency. To determine the group differences between HV and SZ in FCT-DT consistency, a two-sample t-test was performed for 40 WM tracts. Sex, age, education, and mean framewise displacement (FD) of fMRI were considered covariates. The significance level was established at *P* < 0.05 with FDR correction. The effect size was evaluated using Cohen’s d value. In addition, partial correlations were used to test the relationship between FCT-DT consistency in the WM tracts and clinical symptoms. Sex, age, education, and mean FD were included as covariates. FDR correction was used for multiple comparisons; and uncorrected *P* values were also reported in this exploratory study. All data and code used in the current study are available at https://www.dropbox.com/sh/qhch3x537uymr3c/AADojEGtUKByFM6z4NCmRNIOa?dl=0.

## Results

### Demographic and clinical characteristics

In total, 75 individuals with schizophrenia (37 females; age: 42.44 ± 11.04 years) and 89 healthy volunteers (50 females; age: 39.61 ± 12.62 years) were included for analysis in this study. The demographic and clinical characteristics of these subjects were shown in Table 1. No significant differences were found between the two groups in age (*P* = 0.13), sex (*P* = 0.52) and education (*P* = 0.09). As shown in Table 1, the MMSE score in SZ group was significantly lower than in HV group (*P* = 0.002).

### Validation of FCT-DT consistency

To test the reliability of FCT-DT consistency, randomized validations were performed by comparing the FCT-DT consistency between voxels in a specific WM tract and voxels averaged from the GM in the HV group. Compared to the voxels chosen from GM, the voxels in all WM tracts derived from the WM JHU atlas showed significantly stronger structure-function coupling except two tracts (right posterior limb of internal capsule and left tapetum; Supplementary Fig. 1 and Supplementary Table 2). Besides, the internal consistency of FCT-DT consistency was examined by comparing group differences within HV group. For within-group comparison in HV group, there was no significant difference in FCT-DT consistency for all WM regions (all *P* > 0.94).

### Group differences in the WM tracts estimated by FCT-DT consistency

**Fig. 2:**
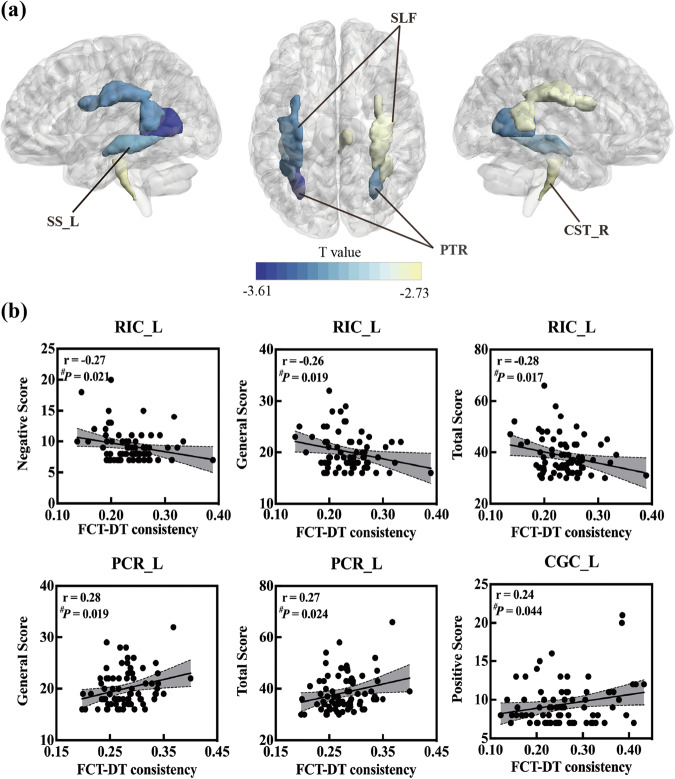
The group differences in FCT-DT consistency in the WM tracts and its correlation to clinical symptoms. **a** The abnormal WM tracts in SZ group. The color of WM tracts corresponds to the T value. SS_L left sagittal stratum, SLF superior longitudinal fasciculus, PTR posterior thalamic radiation, CST_R right corticospinal tract. **b** The correlation between FCT-DT consistency and clinical symptoms in SZ. Partial correlation was used with controlling sex, age, education and mean FD of fMRI. The significance level was set at *P* < 0.05 (uncorrected). RIC_L left retrolenticular part of internal capsule, PCR_L left posterior corona radiata, CGC_L left cingulum (cingulate gyrus), #: uncorrected.

FCT-DT consistency showed significant differences between HV and SZ groups in six WM tracts (Fig. 2a, Supplementary Fig. 2). Overall, all six WM tracts exhibited decreased FCT-DT consistency in the SZ group compared to the HV group. Specifically, reductions in FCT-DT consistency have been observed in the SZ group in bilateral posterior thalamic radiation (left: FDR_corrected *P* = 0.016, *t* = -3.61, Cohen’s d = 0.66; right: FDR_corrected *P* = 0.017, *t* = –3.29, Cohen’s d = 0.50), right corticospinal tract (FDR_corrected *P* = 0.047, *t* = –2.73, Cohen’s d = 0.53), bilateral superior longitudinal fasciculus (left: FDR_corrected *P* = 0.017, *t* = –3.29, Cohen’s d = 0.54; right: FDR_corrected *P* = 0.047, *t* = –2.79, Cohen’s d = 0.36) and left sagittal stratum (FDR_corrected *P* = 0.018, *t* = –3.17, Cohen’s d = 0.57). The uncorrected P value of all WM tracts can be seen in the supplementary table 3.

### The association between FCT-DT consistency and clinical variables in SZ

No correlation can survive after multiple comparison corrections. However, in this exploratory analysis, the trend toward possible correlations between FCT-DT consistency and clinical variables were observed. As shown in Fig. 2b, negative correlations were observed between the FCT-DT consistency in the left retrolenticular part of internal capsule and negative (r = –0.27, *P* = 0.021, uncorrected), general (r = –0.26, *P* = 0.019, uncorrected) and total scores of PANSS (r = –0.28, *P* = 0.017, uncorrected). The FCT-DT consistency in the left posterior corona radiata was found to be positively correlated with general (r = 0.28, *P* = 0.019, uncorrected) and total scores (r = 0.27, *P* = 0.024, uncorrected). The FCT-DT consistency in the cingulum (cingulate gyrus) was found to be positively correlated with positive scores (r = 0.24, *P* = 0.044, uncorrected). The duration of illness was negatively related to the FCT-DT consistency in the right anterior corona radiata and the left posterior corona radiata (Supplementary Fig. 3). The medication was negatively associated with FCT-DT consistency in the external capsule and body of callosum corpus (Supplementary Fig. 4).

## Discussion

In this study, the FCT-DT consistency was proposed as a novel measurement to assess the structure-function coupling in WM. The remarkable increase in FCT-DT consistency in WM compared to those in GM confirmed its potential to delineate the link between function and the underlying structure. Our results revealed that individuals with schizophrenia were characterized by alterations of structure-function coupling in the WM tracts. A significant decrease in the structure-function coupling was found in the right corticospinal tract, bilateral posterior thalamic radiation, bilateral superior longitudinal fasciculus and left sagittal stratum. Furthermore, the structure-function coupling in the WM tracts was found to be related to psychotic symptoms and illness duration in schizophrenia.

The association between structure and function is a fundamental characteristic that reflects the integrity of neural signals [32], which relates to development, aging, cognition and disease [43–48]. Regarding the correspondence between brain structure and function, structural features are primarily measured by fiber counts/intensity or FA values via diffusion metrics, which provide information about anatomical architecture of WM, but are relatively static and cannot reflect information transmission. Functional connectivity is measured by focal activations of voxels or temporal correlations between different regions’ time series, reflecting transient synchronization between cortical areas, but lacking support for whether these areas are structurally connected. In this regard, FCT could reflect the propagation of information because it captured directional variation of resting-state correlations with neighboring regions [19]. Further experiments demonstrated that functional tasks could induce anisotropic correlations along long-range fiber tracts that were absent in the resting state, suggesting that blood-oxygen-level-dependent effects may be driven by neural activity along fiber tracts [20]. According to these observations, it appears that FCT provides the ability to visualize functional pathways based on WM structure. DT can describe the magnitude and orientation of diffusion anisotropy, which is powerful for characterizing the architecture and organization of microstructure [49]. Therefore, combining FCT and DT could provide comprehensive information on the structural characteristic and signal transfer in these structural connections, thereby may facilitate direct identification of the relationship between WM structure and function. By randomized validation, our study demonstrated that FCT-DT consistency in WM was considerably greater than those in GM, which supported the hypothesis that the measurement was able to reflect information on neural signal transfer along WM tracts. WM structural and functional abnormalities have been found in schizophrenia research by means of DTI and fMRI, respectively [8, 11, 16–18]. The association between them, however, is not well explored. According to a previous study, functional changes in WM should be assessed based on structure first [20]. Therefore, applying the proposed FCT-DT consistency to schizophrenia could provide a plausible opportunity for studying neural information propagation along WM tracts.

In the current study, six WM tracts exhibited significantly lower structure-function coupling than those in HV group, indicating the dysconnectivity of schizophrenia with aberrant connectivity between brain regions in the aspect of circuit function. More specifically, a decrease in structure-function coupling was observed in the right corticospinal tract, which is the most important pyramid pathway in the brain responsible for motor function. Motor function impairment in schizophrenia has been examined in several fMRI studies, concluding that motor dysfunction could be an intermediate phenotype candidate for schizophrenia [50–53]. Our results suggested that a reduction in the structure-function coupling in the corticospinal tract may contribute to motor dysfunction in schizophrenia. Here, we demonstrated that posterior thalamic radiation and superior longitudinal fasciculus exhibited decreased structure-function coupling, both related to visuospatial function. The superior longitudinal fasciculus plays an important role in integrating visual stimuli and influencing emotional responses [54]. In addition, the superior longitudinal fasciculus is a prominent associative fiber bundle connecting wide areas of the frontal and parietal cortex, which is related to language function, motor regulation and visuospatial processing [55]. Decreased FA values were found in these regions in a large-sample schizophrenia study [8]. Taken together, we speculated that these abnormal neural circuits in schizophrenia may contribute to the dysfunction of visuospatial attention, motor and language [56–60]. Several recent studies have found converging evidence that spatial variability in the structure-function coupling aligned with cortical hierarchies of functional specialization [45, 46, 61–63]. Previous studies exploring WM functional networks have found that schizophrenia showed increased functional connectivity in the superficial perception-motor network and impaired interactions between networks [11, 16]. A recent research reported that the nodal efficiency of posterior thalamic radiation and sagittal stratum was reduced, indicating lower local information communication in schizophrenia [17]. In our study, fiber bundles showing decreased structure-function coupling were related to functionally segregated regions (e.g., corticospinal tract and posterior thalamic radiation) and functionally integrated regions (e.g., superior longitudinal fasciculus and sagittal stratum). Together, these findings indicated that functional segregation and integration in WM was disrupted in schizophrenia.

Several studies have reported the correlation between structure-function coupling and the severity of symptoms [28, 31]. The lack of association with psychotic symptoms in our study may be due to many factors, including the complicated association between brain connectivity, antipsychotic treatment, clinical manifestation, and a relatively small sample size. One possible explanation is that connectome abnormalities might reflect a potential vulnerability factor for the disease and be more related to aspects of global outcome of schizophrenia rather than symptom severity [27]. Li et al. suggested that structural and functional abnormalities contributed independently to the pathophysiology of schizophrenia [64]. Therefore, combining structural and functional features may complicate the underlying association with clinical variables. Nevertheless, it is worth noting that while the current study did not find a significant association between structure-function coupling and schizophrenia symptoms after multiple comparison corrections, it reported a possible effect. To avoid type II errors, future studies should examine this association in larger sample sizes. Altogether, it has been suggested that structure-function coupling anomalies may not only be an indicator of illness, but also cumulative medication exposure during chronic course [18]. However, the relationship between medication and structure-function coupling is complex due to the different types of drugs that people have taken, whose effect may vary on brain structure and function [65]. Thus, further studies examining the effect of medication therapy with a single antipsychotic drug on the structure-function coupling in schizophrenia may be helpful. For instance, in a study using risperidone monotherapy, Zong et al. found that no change in structure-function coupling pretreatment, whereas it decreased after treatment in drug-naïve first-episode schizophrenia [66]. These findings imply that the decrease in the structure-function coupling was due to antipsychotic treatment to some extent.

This study has several potential limitations. First, most individuals with schizophrenia recruited in this study were chronic and mildly ill (mean illness duration = 16.3 years; mean total PANSS scores = 39.0). Different findings might be observed in other stages of schizophrenia, such as high-risk subjects or first-episode psychosis. Second, each individual with schizophrenia received distinct antipsychotic treatment. It is difficult to differentiate the effect of medication and disease itself on the structure-function coupling of brain networks in individuals with schizophrenia. Third, the tensor model is prone to problems with crossing fiber tracts, which can only represent a single major fiber direction per voxel [67]. Thus, the structure-function coupling evaluated by the FCT-DT consistency was less reliable in these crossing fibers. Fourth, the sample size in this study was relatively small. It might partly explain why the correlation between FCT-DT consistency and psychiatric symptoms failed to pass the multiple comparison corrections. The larger sample size will improve the significance of FCT-DT consistency. Besides, the underlying biological substrate of the structure-function coupling has not yet been well characterized. Finally, we also note that FCT was estimated throughout the fMRI scans, potentially neglecting its temporal dynamics. Future research using time-resolved FCT would provide more information about the change in structure-function coupling over time.

In summary, to the best of our knowledge, this is the first time the FCT-DT consistency has been proposed and used to estimate the structure-function coupling in schizophrenia. According to the randomized validation, the FCT-DT consistency in WM is much higher than that in GM, suggesting that it may reflect neural activity along WM tracts. The proposed measurement might have potential to reveal the activated structural connections underlying functional connectivity between distinct brain regions. On the other hand, widespread decreases in the structure-function coupling within WM tracts in schizophrenia supported the dysconnectivity hypothesis of schizophrenia from the perspective of WM function. These results implied that functional abnormality between GM regions may be caused by aberrant information propagation of neural signals along WM tracts. Furthermore, the association between clinical symptoms and structure-function coupling in the WM tracts suggested that abnormal structure-function coupling may be one of the potential mechanisms of the neuropathology of schizophrenia. As FCT can be modulated by external stimuli [20], the FCT-DT consistency may also be induced and further provides the activated neural pathways in brain activity. A previous motor training study supports such hypothesis by showing significant increases in correlations between FA values of DTI and FCT in the corpus callosum after training [68]. Taken together, these findings highlighted the vital role of structure-function coupling in the WM tracts in the pathophysiology of schizophrenia. Further analysis on FCT-DT consistency in the fiber bundles connecting distinct brain regions may facilitate understanding the role of WM functional circuit in schizophrenia for future studies.

## Supplementary information

Supplementary materials
