# Supplementary material for: Structure-function coupling in white matter uncovers the abnormal brain connectivity in Schizophrenia
Source: Transl Psychiatry. 2023 Jun 21;13:214. doi: 10.1038/s41398-023-02520-4 (PMC10281980; doi:10.1038/s41398-023-02520-4)
Supplement: Supplementary file 1 — Supplementary materials [file 41398_2023_2520_MOESM1_ESM.docx]

**Supplementary Materials**

**Methods**

***Participants exclusion criteria***

Following the imaging quality control procedures (Hagler et al., 2019), 43 subjects were excluded due to incompleteness of the imaging data, motion, artefacts, poor signal-to-noise ratio or poor gray/white contrast. To be specific, four subjects with incomplete rs-fMRI or DWI images scans were removed (incomplete rs-fMRI images: 1 HV; incomplete DWI images: 1 SZ, 2 HV); Five participants who only had rs-fMRI or DWI images were excluded (missing rs-fMRI: 1 SZ; missing DWI: 4 SZ); Fifteen subjects with motion parameters obtained from rs-fMRI exceeded the predefined threshold (8 SZ, 7 HV). Eighteen subjects with poor image quality (such as motion, low SNR, poor CNR, signal loss) of DWI or fMRI scans were excluded (13SZ, 5HV). Besides, one subject was excluded due to severe depression symptoms (1SZ). Finally, the final sample included 75 SZ and 89 HV in this study.

***Construction of functional correlation tensor***

For each voxel, the FCT was constructed to characterize the local profiles of temporal correlation between the voxel and its neighbors. The FCT, $T_{i}$ for voxel $i$ can be represented by a 3 × 3 symmetric matrix as follows (Ding et al., 2016):

$$T_{i}=\left[ \begin{matrix} T_{xx} & T_{xy} & T_{xz} \\ T_{xy} & T_{yy} & T_{yz} \\ T_{xz} & T_{yz} & T_{zz} \end{matrix} \right]$$

Mathematically, the $T_{i}$ was obtained using the following steps:

1) a unit direction vector $n_{ij}=(x_{ij},y_{ij},z_{ij})$ connecting a given voxel $i$ and its neighbors $j$, $j\in\{1,2,\ldots26\}$.

2) the correlation coefficient $C_{ij}$ of time series between a given voxel $i$ and its neighbors $j$, $j\in\{1,2,\ldots26\}$.

3) the relationship between $C_{ij}$ and $T_{i}$ of a given voxel $i$ is denoted as:

$C_{i}=M_{i}\cdot\bar{T_{i}}$,

where $C_{i}=\left[ C_{i,1},C_{i,2},\ldots C_{i,26} \right]^{t}$. $M_{i}$ is a design matrix of size 26 × 6. The $j^{th}$row of $M_{i}$ has the form of $({x_{ij}}^{2},2x_{ij}y_{ij},2x_{ij}z_{ij},{y_{ij}}^{2},2y_{ij}z_{ij},{z_{ij}}^{2})$. $\bar{T_{i}}$ is a column vector rearranged from $T_{i}$, $\bar{T_{i}}=\left[ T_{xx},T_{xy},T_{xz},T_{yy},T_{yz},T_{zz} \right]^{t}$.$t$ denotes a transpose operation.

4) a least-square solution for $\bar{T_{i}}$can be obtained as follows:

$$\bar{T_{i}}=\left( {M_{i}}^{t}\cdot M_{i} \right)^{-1}\cdot{M_{i}}^{t}\cdot C_{i}$$

where -1 denotes matrix inverse.

Here, we computed the FCT of all voxels in WM using rs-fMRI for all subjects.

***Validation of FCT-DT consistency***

To validate the nonrandom organization of FCT-DT consistency in the WM tracts, the mean FCT-DT consistency in voxels in a specific WM tract was compared to the mean FCT-DT consistency in voxels from GM. All 40 WM tracts in native DWI space were included for analysis. The FCT-DT consistency in a specific WM tract (for example, left corticospinal tract) was calculated by averaging all consistency values from voxels belonging to this tract. The GM acquired from brain segmentation was also registered to DWI image and only voxels with intensity value greater than 0.7 were retained (Johnson et al., 2013). The FCT-DT consistency in GM was the mean value of calculated consistency values in these retained voxels. Besides, in order to examine the internal consistency of FCT-DT consistency, the HV group was randomly divided into two halves and repeated 5000 times. The subgroup comparisons in FCT-DT consistency were made for all WM tracts.

**Supplementary Results
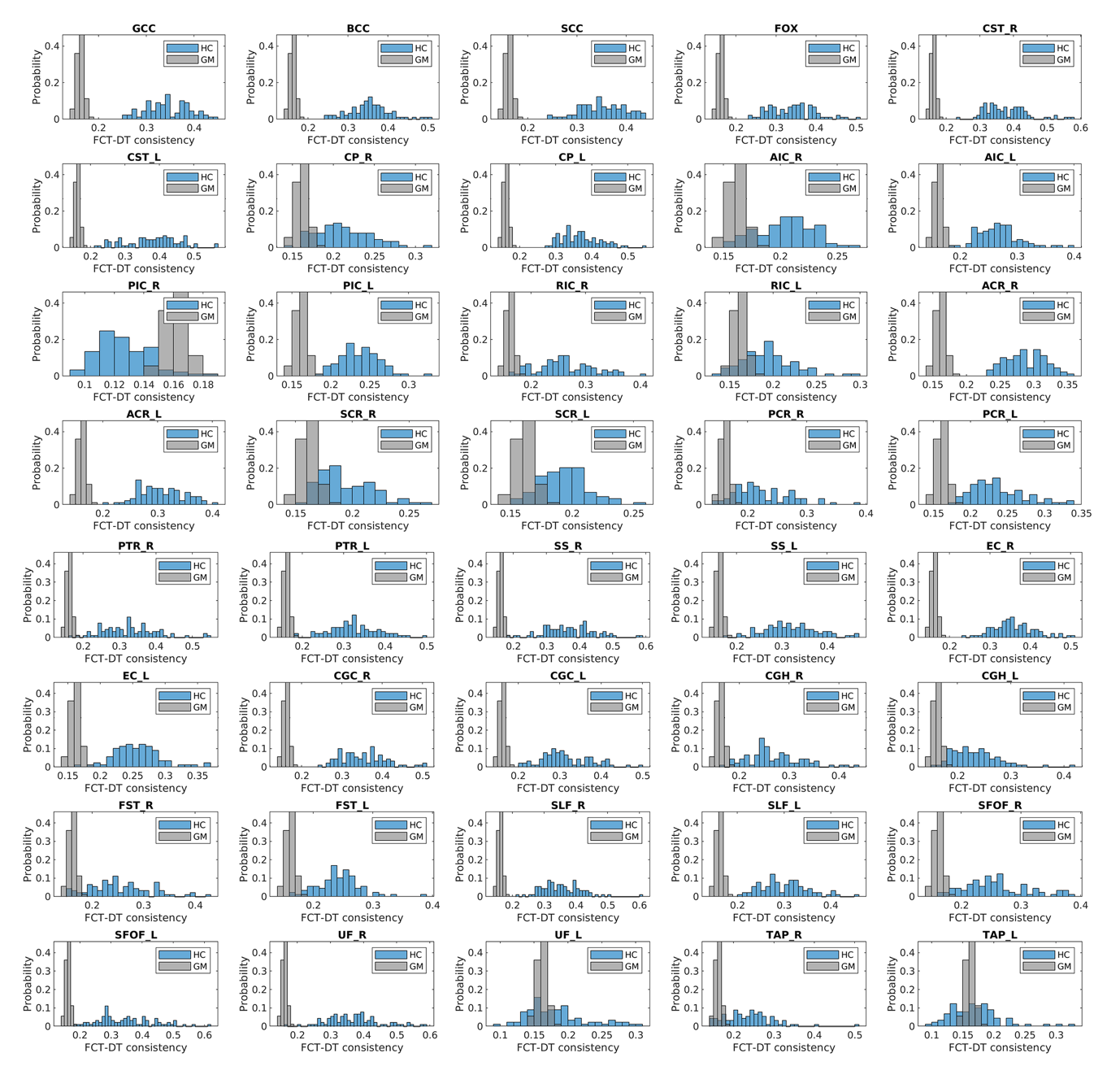
**

**Supplementary Figure 1. The distribution of FCT-DT consistency in voxels from WM tracts and GM.** HC：healthy controls, GM: gray matter. See supplementary Table 4 for a summary of full name of each WM tract.

**
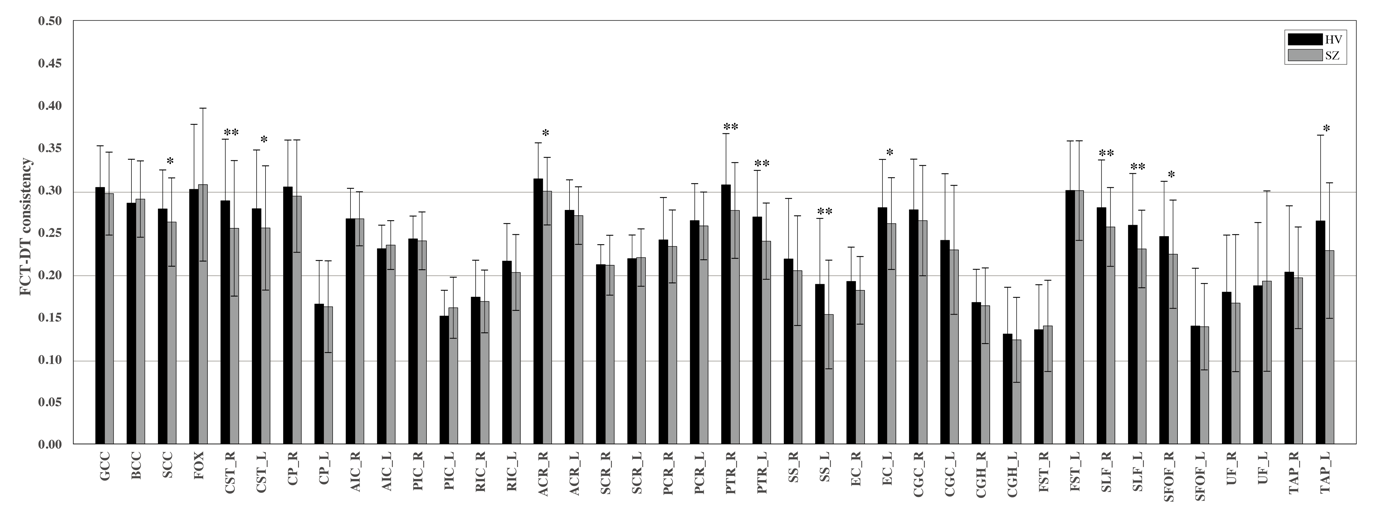
Supplementary Figure 2. Comparison of FCT-DT consistency in WM between SZ and HV group.** * indicates P < 0.05 uncorrected. ** indicates P < 0.05 with FDR correction. SZ, schizophrenia; HV, healthy controls. See supplementary Table 4 for a summary of full name of each WM tract.

**
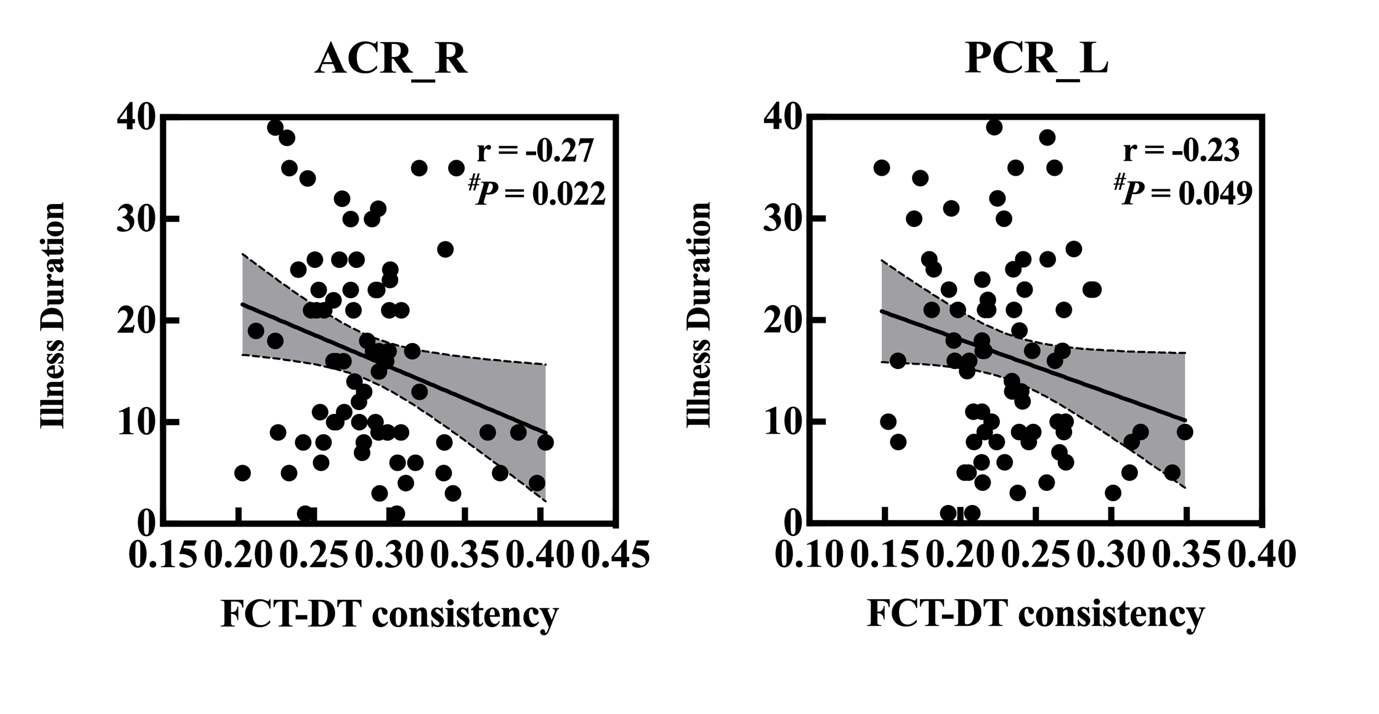
**

**Supplementary Figure 3. The correlation of FCT-DT consistency in the WM tracts and illness duration.** ACR_R: right anterior corona radiata, PCR_L: left posterior corona radiata, ^#^: uncorrected.

**
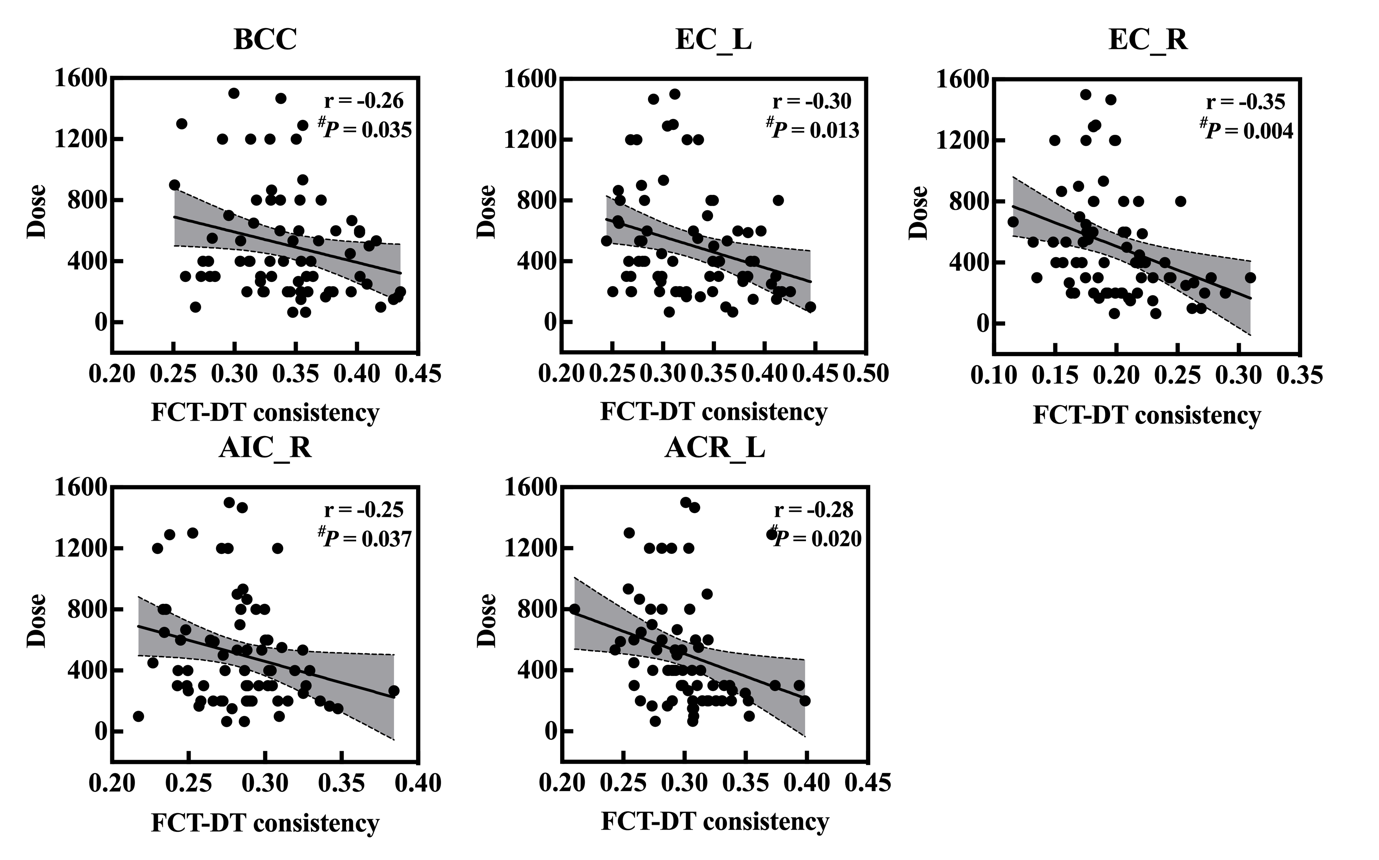
Supplementary Figure 4. The correlation of FCT-DT consistency in the WM tracts and medication.** BCC: body of callosum corpus, EC_L: left external capsule, EC_R: right external capsule, AIC_R: right anterior limb of internal capsule, ACR_L: left anterior corona radiata, ^#^: uncorrected.

**Supplementary Table 1. The dosage information for each subject with schizophrenia.**

| **ID** | **Primary Antipsychotics** | **Dosage(mg)** | **CPZ** |
| --- | --- | --- | --- |
| MRSZ001 | Clozapine | 100 | 266.7 |
| MRSZ002 | Risperidone | 6 | 300 |
| MRSZ003 | Risperidone | 4 | 200 |
| MRSZ004 | Olanzapine | 20 | 400 |
| MRSZ005 | Olanzapine | 20 | 400 |
| MRSZ006 | Invega | 6 | 400 |
| MRSZ007 | Solian | 800 | 1800 |
| MRSZ008 | Clozapine | 350 | 700 |
| MRSZ010 | Clozapine | 250 | 589.3 |
| MRSZ012 | Sulpiride | 400 | 200 |
| MRSZ015 | Haldol Decanoas | 50 | 166.7 |
| MRSZ018 | Olanzapine | 20 | 400 |
| MRSZ019 | Haldol | 5 | 250 |
| MRSZ020 | Clozapine | 150 | 300 |
| MRSZ023 | Olanzapine | 20 | 550 |
| MRSZ024 | Clozapine | 200 | 533.3 |
| MRSZ026 | Seroquel | 900 | 1600 |
| MRSZ027 | Seroquel | 700 | 933.3 |
| MRSZ028 | Clozapine | 200 | 1200 |
| MRSZ029 | Solian | 400 | 900 |
| MRSZ030 | Clozapine | 400 | 800 |
| MRSZ031 | Clozapine | 250 | 500 |
| MRSZ032 | Clozapine | 200 | 533.3 |
| MRSZ033 | Risperidone | 4 | 200 |
| MRSZ034 | Olanzapine | 5 | 100 |
| MRSZ035 | Olanzapine | 10 | 200 |
| MRSZ036 | Clozapine | 300 | 600 |
| MRSZ037 | Solian | 400 | 200 |
| MRSZ038 | Clozapine | 600 | 1466.7 |
| MRSZ039 | Invega | 6 | 400 |
| MRSZ040 | Olanzapine | 20 | 600 |
| MRSZ042 | Fluanxol | 20 | 666.7 |
| MRSZ043 | Seroquel | 300 | 533.3 |
| MRSZ044 | Olanzapine | 10 | 200 |
| MRSZ046 | Risperidone | 3 | 150 |
| MRSZ047 | Invega Sustenna | 100 | 600 |
| MRSZ049 | Risperidone | 4 | 200 |
| MRSZ052 | Clozapine | 400 | 800 |
| MRSZ053 | Solian | 400 | 800 |
| MRSZ061 | Fluanxol | 6 | 200 |
| MRSZ063 | Sulpiride | 50 | 25 |
| MRSZ066 | Morefine | 200 | 450 |
| MRSZ067 | Olanzapine | 7.5 | 150 |
| MRSZ068 | Clozapine | 100 | 300 |
| MRSZ071 | Sulpiride | 400 | 200 |
| MRSZ072 | Abilify | 15 | 200 |
| MRSZ073 | Risperidone | 6 | 300 |
| MRSZ074 | Clozapine | 600 | 1300 |
| MRSZ077 | Solian | 600 | 1200 |
| MRSZ079 | Invega | 9 | 1200 |
| MRSZ080 | Olanzapine | 20 | 400 |
| MRSZ082 | Haldol Decanoas | 50 | 166.7 |
| MRSZ083 | Clozapine | 300 | 866.7 |
| MRSZ090 | Solian | 150 | 300 |
| MRSZ092 | Olanzapine | 10 | 200 |
| MRSZ093 | Olanzapine | 22.5 | 650 |
| MRSZ094 | Clozapine | 200 | 533.3 |
| MRSZ095 | Solian | 400 | 800 |
| MRSZ097 | Clozapine | 150 | 1500 |
| MRSZ098 | Olanzapine | 20 | 400 |
| MRSZ100 | Solian | 200 | 400 |
| MRSZ103 | Risperidone | 2 | 100 |
| MRSZ104 | Risperidone | 6 | 300 |
| MRSZ105 | Etumine | 80 | 800 |
| MRSZ107 | Solian | 800 | 1600 |
| MRSZ119 | Olanzapine | 15 | 300 |
| MRSZ126 | Olanzapine | 20 | 1200 |
| MRSZ130 | Invega | 18 | 1289.3 |
| MRSZ131 | Abilify | 20 | 266.7 |
| MRSZ133 | Olanzapine | 10 | 200 |
| MRSZ144 | Abilify | 5 | 66.7 |
| MRSZ145 | Sulpiride | 600 | 300 |
| MRSZ146 | Olanzapine | 15 | 300 |
| MRSZ156 | Abilify | 5 | 66.7 |
| MRSZ161 | Clozapine | 200 | 400 |
| MRSZ168 | Abilify | 30 | 400 |
| MRSZ171 | Clozapine | 300 | 600 |
| MRSZ175 | #N/A | #N/A | #N/A |
| MRSZ179 | #N/A | #N/A | #N/A |
| MRSZ184 | #N/A | #N/A | #N/A |

**Supplementary Table 2. The group differences of FCT-DT consistency in voxels from WM tracts and GM.**

| **Label** | ***P*_FDR** | **Tvalue** | **Label** | ***P*_FDR** | **Tvalue** |
| --- | --- | --- | --- | --- | --- |
| GCC | <0.0001 | 37.69 | PTR_R | <0.0001 | 20.43 |
| BCC | <0.0001 | 33.93 | PTR_L | <0.0001 | 25.42 |
| SCC | <0.0001 | 39.20 | SS_R | <0.0001 | 25.52 |
| FOX | <0.0001 | 29.36 | SS_L | <0.0001 | 24.46 |
| CST_R | <0.0001 | 31.20 | EC_R | <0.0001 | 34.73 |
| CST_L | <0.0001 | 26.38 | EC_L | <0.0001 | 24.45 |
| CP_R | <0.0001 | 13.92 | CGC_R | <0.0001 | 31.36 |
| CP_L | <0.0001 | 35.69 | CGC_L | <0.0001 | 23.99 |
| AIC_R | <0.0001 | 15.87 | CGH_R | <0.0001 | 18.45 |
| AIC_L | <0.0001 | 26.31 | CGH_L | <0.0001 | 15.06 |
| **PIC_R** | **<0.0001** | **-17.73** | FST_R | <0.0001 | 14.61 |
| PIC_L | <0.0001 | 27.28 | FST_L | <0.0001 | 20.87 |
| RIC_R | <0.0001 | 18.07 | SLF_R | <0.0001 | 28.10 |
| RIC_L | <0.0001 | 9.63 | SLF_L | <0.0001 | 25.57 |
| ACR_R | <0.0001 | 39.52 | SFOF_R | <0.0001 | 16.75 |
| ACR_L | <0.0001 | 32.53 | SFOF_L | <0.0001 | 19.18 |
| SCR_R | <0.0001 | 12.10 | UF_R | <0.0001 | 23.87 |
| SCR_L | <0.0001 | 14.32 | UF_L | <0.0001 | 4.42 |
| PCR_R | <0.0001 | 12.25 | TAP_R | <0.0001 | 11.66 |
| PCR_L | <0.0001 | 19.46 | **TAP_L** | **0.073** | **1.80** |

**Supplementary Table 3. Comparison of FCT-DT consistency in WM between HV and SZ group**.

| **Label** | **HV**  **(mean** ±**sd)** | **SZ**  **(mean** ±**sd)** | ***P value*** | **Label** | **HV**  **(mean** ±**sd)** | **SZ**  **(mean** ±**sd)** | ***P value*** |
| --- | --- | --- | --- | --- | --- | --- | --- |
| GCC | 0.30±0.05 | 0.30±0.05 | 0.340 | **PTR_R** | 0.31±0.06 | 0.28±0.06 | 0.001****** |
| BCC | 0.28±0.05 | 0.29±0.05 | 0.540 | **PTR_L** | 0.27±0.05 | 0.24±0.04 | <0.001****** |
| SCC | 0.28±0.05 | 0.26±0.05 | 0.045***** | SS_R | 0.22±0.07 | 0.20±0.06 | 0.199 |
| FOX | 0.30±0.08 | 0.31±0.09 | 0.672 | **SS_L** | 0.19±0.08 | 0.15±0.06 | 0.002****** |
| **CST_R** | 0.29±0.07 | 0.25±0.08 | 0.007****** | EC_R | 0.19±0.04 | 0.18±0.04 | 0.095 |
| CST_L | 0.28±0.07 | 0.26±0.07 | 0.043***** | EC_L | 0.28±0.06 | 0.26±0.05 | 0.032***** |
| CP_R | 0.30±0.06 | 0.29±0.07 | 0.255 | CGC_R | 0.28±0.06 | 0.26±0.07 | 0.192 |
| CP_L | 0.17±0.05 | 0.16±0.05 | 0.702 | CGC_L | 0.24±0.08 | 0.23±0.08 | 0.353 |
| AIC_R | 0.27±0.04 | 0.27±0.03 | 0.995 | CGH_R | 0.17±0.04 | 0.16±0.04 | 0.545 |
| AIC_L | 0.23±0.03 | 0.23±0.03 | 0.338 | CGH_L | 0.13±0.06 | 0.12±0.05 | 0.405 |
| PIC_R | 0.24±0.03 | 0.24±0.03 | 0.618 | FST_R | 0.13±0.05 | 0.14±0.05 | 0.599 |
| PIC_L | 0.15±0.03 | 0.16±0.04 | 0.063 | FST_L | 0.30±0.06 | 0.30±0.06 | 0.981 |
| RIC_R | 0.17±0.04 | 0.17±0.04 | 0.422 | **SLF_R** | 0.28±0.06 | 0.26±0.05 | 0.006****** |
| RIC_L | 0.22±0.04 | 0.20±0.04 | 0.055 | **SLF_L** | 0.26±0.06 | 0.23±0.05 | 0.001****** |
| ACR_R | 0.31±0.04 | 0.30±0.04 | 0.025***** | SFOF_R | 0.25±0.07 | 0.22±0.06 | 0.040***** |
| ACR_L | 0.28±0.04 | 0.27±0.03 | 0.247 | SFOF_L | 0.14±0.07 | 0.14±0.05 | 0.926 |
| SCR_R | 0.21±0.02 | 0.21±0.04 | 0.866 | UF_R | 0.18±0.07 | 0.17±0.08 | 0.263 |
| SCR_L | 0.22±0.03 | 0.22±0.03 | 0.805 | UF_L | 0.19±0.07 | 0.19±0.11 | 0.702 |
| PCR_R | 0.24±0.05 | 0.23±0.04 | 0.292 | TAP_R | 0.20±0.08 | 0.20±0.06 | 0.541 |
| PCR_L | 0.26±0.04 | 0.26±0.04 | 0.338 | TAP_L | 0.26±0.1 | 0.23±0.08 | 0.016***** |

Note * *P* < 0.05 uncorrected, ** *P* < 0.05 with passing FDR correction.

**Supplementary Table 4. The full name of 40 WM labels.**

| **Abbreviation** | **Full Name** |
| --- | --- |
| GCC | Genu of corpus callosum |
| BCC | Body of corpus callosum |
| SCC | Splenium of corpus callosum |
| FOX | Fornix |
| CST_R | Corticospinal tract R |
| CST_L | Corticospinal tract L |
| CP_R | Cerebral peduncle R |
| CP_L | Cerebral peduncle L |
| AIC_R | Anterior limb of internal capsule R |
| AIC_L | Anterior limb of internal capsule L |
| PIC_R | Posterior limb of internal capsule R |
| PIC_L | Posterior limb of internal capsule L |
| RIC_R | Retrolenticular part of internal capsule R |
| RIC_L | Retrolenticular part of internal capsule L |
| ACR_R | Anterior corona radiata R |
| ACR_L | Anterior corona radiata L |
| SCR_R | Superior corona radiata R |
| SCR_L | Superior corona radiata L |
| PCR_R | Posterior corona radiata R |
| PCR_L | Posterior corona radiata L |
| PTR_R | Posterior thalamic radiation R |
| PTR_L | Posterior thalamic radiation L |
| SS_R | Sagittal stratum R |
| SS_L | Sagittal stratum L |
| EC_R | External capsule R |
| EC_L | External capsule L |
| CGC_R | Cingulum (cingulate gyrus) R |
| CGC_L | Cingulum (cingulate gyrus) L |
| CGH_R | Cingulum (hippocampus) R |
| CGH_L | Cingulum (hippocampus) L |
| FST_R | Fornix (cres) / Stria terminalis R |
| FST_L | Fornix (cres) / Stria terminalis L |
| SLF_R | Superior longitudinal fasciculus R |
| SLF_L | Superior longitudinal fasciculus L |
| SFOF_R | Superior fronto-occipital fasciculus R |
| SFOF_L | Superior fronto-occipital fasciculus L |
| UF_R | Uncinate fasciculus R |
| UF_L | Uncinate fasciculus L |
| TAP_R | Tapetum R |
| TAP_L | Tapetum L |

**References**

Ding, Z., Xu, R., Bailey, S. K., Wu, T. L., Morgan, V. L., Cutting, L. E., . . . Gore, J. C. (2016). Visualizing functional pathways in the human brain using correlation tensors and magnetic resonance imaging. *Magn Reson Imaging*, *34*(1), 8-17. <https://doi.org/10.1016/j.mri.2015.10.003>

Hagler, D. J., Jr., Hatton, S., Cornejo, M. D., Makowski, C., Fair, D. A., Dick, A. S., . . . Dale, A. M. (2019). Image processing and analysis methods for the Adolescent Brain Cognitive Development Study. *NeuroImage*, *202*, 116091. <https://doi.org/10.1016/j.neuroimage.2019.116091>

Johnson, C. L., McGarry, M. D., Gharibans, A. A., Weaver, J. B., Paulsen, K. D., Wang, H., . . . Georgiadis, J. G. (2013). Local mechanical properties of white matter structures in the human brain. *NeuroImage*, *79*, 145-152. <https://doi.org/10.1016/j.neuroimage.2013.04.089>
